# 3-Acetyl-6-chloro-2-methyl-4-phenyl­quinolinium perchlorate

**DOI:** 10.1107/S1600536810012900

**Published:** 2010-04-28

**Authors:** Tara Shahani, Hoong-Kun Fun, S. Sarveswari, V. Vijayakumar, B. Palakshi Reddy

**Affiliations:** a X-ray Crystallography Unit, School of Physics, Universiti Sains Malaysia, 11800 USM, Penang, Malaysia; b Organic Chemistry Division, School of Advanced Sciences, VIT University, Vellore 632 014, India

## Abstract

In the title mol­ecular salt, C_18_H_15_ClNO^+^·ClO_4_^−^, the quinolin­ium ring system is approximately planar, with a maximum deviation of 0.027 (1) Å. The dihedral angle formed between the mean planes of the quinolinium ring system and the benzene ring is 78.46 (3)°. In the crystal structure, inter­molecular N—H⋯O and C—H⋯O hydrogen bonds link the cations and anions into a three-dimensional network. The crystal structure is further consolidated by C—H⋯π inter­actions.

## Related literature

For natural products containing quinolines, see: Michael (1997 ▶); Morimoto *et al.* (1991 ▶). For the biological activities of quinolines, see: Campbell *et al.* (1988 ▶); Markees *et al.* (1970 ▶). For the physiological activities of quinolines, see: Katritzky & Arend (1998 ▶); Jiang & Si (2002 ▶). For related structures, see: Shahani *et al.* (2010 ▶); Fun *et al.* (2009 ▶); Loh *et al.* (2010 ▶). For bond-length data, see: Allen *et al.* (1987 ▶). For the stability of the temperature controller used for the data collection, see: Cosier & Glazer (1986 ▶).
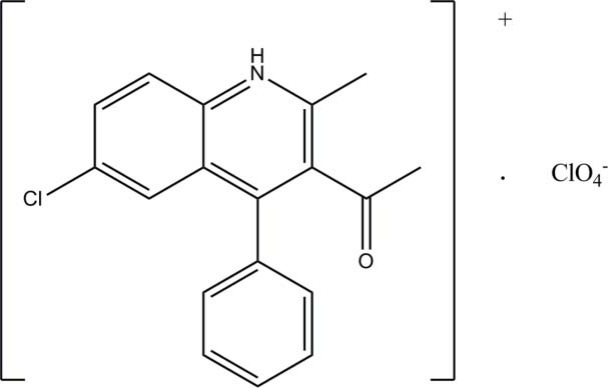


## Experimental

### 

#### Crystal data

C_18_H_15_ClNO^+^·ClO_4_^−^
*M* *_r_* = 396.21
Triclinic, 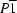

*a* = 7.3862 (1) Å
*b* = 8.8519 (2) Å
*c* = 13.3378 (3) Å
α = 92.477 (1)°
β = 91.903 (1)°
γ = 99.550 (1)°
*V* = 858.44 (3) Å^3^
*Z* = 2
Mo *K*α radiation
μ = 0.41 mm^−1^
*T* = 100 K
0.58 × 0.54 × 0.27 mm

#### Data collection

Bruker SMART APEXII CCD diffractometer
Absorption correction: multi-scan (*SADABS*; Bruker, 2009 ▶) *T*_min_ = 0.797, *T*_max_ = 0.898
27967 measured reflections
7482 independent reflections
6933 reflections with *I* > 2σ(*I*)
*R*_int_ = 0.019

#### Refinement

*R*[*F* ^2^ > 2σ(*F* ^2^)] = 0.031
*wR*(*F* ^2^) = 0.108
*S* = 1.09
7482 reflections
295 parameters
All H-atom parameters refined
Δρ_max_ = 0.69 e Å^−3^
Δρ_min_ = −1.00 e Å^−3^

### 

Data collection: *APEX2* (Bruker, 2009 ▶); cell refinement: *SAINT* (Bruker, 2009 ▶); data reduction: *SAINT*; program(s) used to solve structure: *SHELXTL* (Sheldrick, 2008 ▶); program(s) used to refine structure: *SHELXTL*; molecular graphics: *SHELXTL*; software used to prepare material for publication: *SHELXTL* and *PLATON* (Spek, 2009 ▶).

## Supplementary Material

Crystal structure: contains datablocks global, I. DOI: 10.1107/S1600536810012900/hb5397sup1.cif

Structure factors: contains datablocks I. DOI: 10.1107/S1600536810012900/hb5397Isup2.hkl

Additional supplementary materials:  crystallographic information; 3D view; checkCIF report

## Figures and Tables

**Table 1:** Hydrogen-bond geometry (Å, °) *Cg*1 is the centroid of the C10–C15 ring.

*D*—H⋯*A*	*D*—H	H⋯*A*	*D*⋯*A*	*D*—H⋯*A*
N1—H1N1⋯O3^i^	0.832 (18)	1.896 (18)	2.7177 (10)	169 (2)
C3—H3*A*⋯O2^ii^	0.955 (16)	2.583 (16)	3.3010 (11)	132.2 (12)
C15—H15*A*⋯O5	0.951 (16)	2.512 (16)	3.3716 (12)	150.4 (13)
C18—H18*B*⋯O5^iii^	0.97 (2)	2.53 (2)	3.3266 (13)	139.5 (14)
C12—H12*A*⋯*Cg*1^iv^	0.981 (17)	2.694 (17)	3.5810 (10)	150.6 (13)

Symmetry codes: (i) 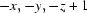; (ii) 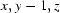; (iii) 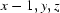; (iv) 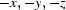.

**Fig. 1.**
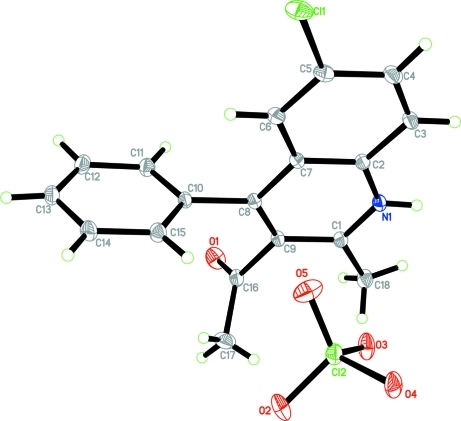
The molecular structure of (I), showing 50% probability displacement ellipsoids.

**Fig. 2.**
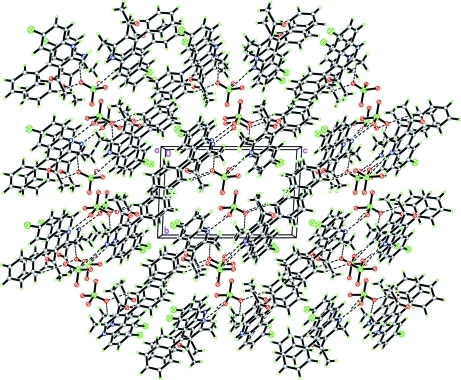
The crystal packing of (I), viewed along a axis. H atoms not involved in intermolecular interactions (dashed lines) are omitted for clarity.

## supplementary crystallographic information

### Comment

Quinolines and their derivatives are very important compounds because of their wide occurrence in natural products (Morimoto *et al.*, 1991; Michael, 1997), and biologically active compounds (Markees *et al.*, 1970 ; Campbell *et al.*, 1988). A large variety of quinolines have interesting physiological activities and found attractive applications as pharmaceuticals, agrochemicals and as synthetic building blocks, due to their great importance, the synthesis of new derivatives of quinoline remains an active research area (Katritzky & Arend, 1998; Jiang & Si, 2002).

In the title compound (Fig. 1), the asymmetric unit consists one perchlorate anion and one 3-acetyl-6-chloro-2-methyl-4-phenlquineline-1-ium cation. The quinolinium ring system (C1/N1/C2–C9) is approximately planar, with a maximum deviation of 0.027 (1) Å at atom C1. The dihedral angle formed between quinolinium ring system and benzene ring (C10–C15) is 78.46 (3)°. Bond lengths (Allen *et al.*, 1987) and angles are normal and comparable to those related structures (Shahani *et al.*, 2010; Fun *et al.*, 2009; Loh *et al.*, 2010).

In the crystal packing (Fig. 2), intermolecular N1—H1N1···O3, C3—H3A···O2, C15—H15A···O5 and C18—H18B···O5 hydrogen bonds (Table 1) link the molecules into three-dimensional network. This crystal structure is further consolidated by C—H···π interactions involving C10–C15 benzene ring (centroid *Cg*1).

### Experimental

A mixture of 3-acetyl-6-chloro-2-methyl-4-phenylquinoline and a catalytic amount of nickel chloride in acid medium was refluxed for about an hour and resultant compound was recrystallized from 3:1 ethanol water to yield colourless blocks of (I).

### Refinement

All H atoms were located in a difference map and was refined freely. [N—H = 0.829 (19) Å, C—H = 0.76 (2)–1.025 (17) Å].

### Crystal data

**Table:**

C_18_H_15_ClNO^+^·ClO_4_^−^	*Z* = 2
*M**_r_* = 396.21	*F*(000) = 408
Triclinic, *P*1	*D*_x_ = 1.533 Mg m^−^^3^
Hall symbol: -P 1	Mo *K*α radiation, λ = 0.71073 Å
*a* = 7.3862 (1) Å	Cell parameters from 9929 reflections
*b* = 8.8519 (2) Å	θ = 2.7–35.1°
*c* = 13.3378 (3) Å	µ = 0.41 mm^−^^1^
α = 92.477 (1)°	*T* = 100 K
β = 91.903 (1)°	Block, colourless
γ = 99.550 (1)°	0.58 × 0.54 × 0.27 mm
*V* = 858.44 (3) Å^3^	

### Data collection

**Table:**

Bruker SMART APEXII CCD diffractometer	7482 independent reflections
Radiation source: fine-focus sealed tube	6933 reflections with *I* > 2σ(*I*)
graphite	*R*_int_ = 0.019
φ and ω scans	θ_max_ = 35.0°, θ_min_ = 1.5°
Absorption correction: multi-scan (*SADABS*; Bruker, 2009)	*h* = −11→11
*T*_min_ = 0.797, *T*_max_ = 0.898	*k* = −13→14
27967 measured reflections	*l* = −21→21

### Refinement

**Table:**

Refinement on *F*^2^	Primary atom site location: structure-invariant direct methods
Least-squares matrix: full	Secondary atom site location: difference Fourier map
*R*[*F*^2^ > 2σ(*F*^2^)] = 0.031	Hydrogen site location: inferred from neighbouring sites
*wR*(*F*^2^) = 0.108	All H-atom parameters refined
*S* = 1.09	*w* = 1/[σ^2^(*F*_o_^2^) + (0.0636*P*)^2^ + 0.2949*P*] where *P* = (*F*_o_^2^ + 2*F*_c_^2^)/3
7482 reflections	(Δ/σ)_max_ < 0.001
295 parameters	Δρ_max_ = 0.69 e Å^−^^3^
0 restraints	Δρ_min_ = −1.00 e Å^−^^3^

### Special details

**Table:**

Experimental. The crystal was placed in the cold stream of an Oxford Cyrosystems Cobra open-flow nitrogen cryostat (Cosier & Glazer, 1986) operating at 100.0 (1) K.
Geometry. All esds (except the esd in the dihedral angle between two l.s. planes) are estimated using the full covariance matrix. The cell esds are taken into account individually in the estimation of esds in distances, angles and torsion angles; correlations between esds in cell parameters are only used when they are defined by crystal symmetry. An approximate (isotropic) treatment of cell esds is used for estimating esds involving l.s. planes.
Refinement. Refinement of *F*^2^ against ALL reflections. The weighted *R*-factor wR and goodness of fit *S* are based on *F*^2^, conventional *R*-factors *R* are based on *F*, with *F* set to zero for negative *F*^2^. The threshold expression of *F*^2^ > σ(*F*^2^) is used only for calculating *R*-factors(gt) etc. and is not relevant to the choice of reflections for refinement. *R*-factors based on *F*^2^ are statistically about twice as large as those based on *F*, and *R*- factors based on ALL data will be even larger.

### Fractional atomic coordinates and isotropic or equivalent isotropic displacement parameters (Å^2^)

**Table:**

	*x*	*y*	*z*	*U*_iso_*/*U*_eq_	
Cl1	0.57962 (3)	−0.15711 (3)	0.127788 (19)	0.01984 (6)	
O1	−0.34868 (10)	0.25202 (9)	0.18488 (6)	0.02125 (14)	
N1	−0.06256 (10)	−0.06546 (8)	0.35547 (5)	0.01256 (12)	
C1	−0.15762 (11)	0.04733 (9)	0.34086 (6)	0.01226 (13)	
C2	0.08539 (11)	−0.08886 (9)	0.30060 (6)	0.01178 (13)	
C3	0.17738 (12)	−0.21217 (10)	0.32169 (6)	0.01442 (14)	
C4	0.32724 (13)	−0.23218 (10)	0.26722 (7)	0.01553 (14)	
C5	0.38693 (12)	−0.12954 (10)	0.19231 (7)	0.01427 (14)	
C6	0.29771 (11)	−0.01015 (9)	0.16974 (6)	0.01300 (13)	
C7	0.14195 (11)	0.01160 (9)	0.22419 (6)	0.01113 (12)	
C8	0.03934 (11)	0.13153 (9)	0.20510 (6)	0.01083 (12)	
C9	−0.10849 (11)	0.14720 (9)	0.26283 (6)	0.01146 (12)	
C10	0.09383 (11)	0.23714 (9)	0.12361 (6)	0.01128 (12)	
C11	−0.00355 (12)	0.21724 (10)	0.03113 (6)	0.01471 (14)	
C12	0.05201 (13)	0.31380 (11)	−0.04601 (7)	0.01646 (15)	
C13	0.20163 (13)	0.43138 (10)	−0.03026 (7)	0.01619 (15)	
C14	0.29697 (13)	0.45204 (10)	0.06228 (7)	0.01649 (15)	
C15	0.24533 (12)	0.35437 (10)	0.13932 (6)	0.01454 (14)	
C16	−0.21861 (12)	0.27461 (10)	0.24424 (6)	0.01340 (13)	
C17	−0.15358 (17)	0.42459 (12)	0.30030 (8)	0.02276 (18)	
C18	−0.31035 (13)	0.06418 (11)	0.40871 (7)	0.01699 (15)	
H3A	0.137 (2)	−0.2799 (18)	0.3733 (12)	0.017 (3)*	
H4A	0.394 (3)	−0.310 (2)	0.2795 (13)	0.029 (4)*	
H6A	0.343 (2)	0.0569 (18)	0.1200 (11)	0.016 (3)*	
H11A	−0.117 (2)	0.1334 (19)	0.0189 (12)	0.022 (4)*	
H12A	−0.013 (2)	0.2955 (19)	−0.1119 (13)	0.023 (4)*	
H13A	0.239 (2)	0.5034 (19)	−0.0772 (12)	0.021 (4)*	
H14A	0.394 (2)	0.5305 (19)	0.0737 (12)	0.021 (4)*	
H15A	0.306 (2)	0.3656 (18)	0.2039 (12)	0.020 (4)*	
H17A	−0.033 (3)	0.460 (2)	0.2780 (14)	0.031 (4)*	
H17B	−0.242 (3)	0.488 (2)	0.2843 (15)	0.037 (5)*	
H17C	−0.153 (3)	0.418 (3)	0.3570 (18)	0.045 (6)*	
H18A	−0.281 (3)	0.154 (3)	0.4486 (16)	0.042 (5)*	
H18B	−0.424 (3)	0.078 (2)	0.3754 (14)	0.031 (4)*	
H18C	−0.334 (3)	−0.026 (2)	0.4431 (15)	0.037 (5)*	
H1N1	−0.089 (3)	−0.121 (2)	0.4034 (14)	0.030 (4)*	
Cl2	0.28102 (3)	0.33444 (2)	0.461521 (15)	0.01541 (5)	
O2	0.27428 (10)	0.49972 (8)	0.45103 (6)	0.02070 (14)	
O3	0.10191 (10)	0.25407 (9)	0.48844 (6)	0.01977 (14)	
O4	0.40805 (10)	0.32400 (9)	0.55505 (5)	0.01960 (13)	
O5	0.35420 (12)	0.26779 (11)	0.37509 (6)	0.02648 (16)	

### Atomic displacement parameters (Å^2^)

**Table:**

	*U*^11^	*U*^22^	*U*^33^	*U*^12^	*U*^13^	*U*^23^
Cl1	0.01521 (10)	0.01761 (10)	0.02749 (11)	0.00551 (7)	0.00402 (7)	−0.00306 (8)
O1	0.0153 (3)	0.0210 (3)	0.0279 (4)	0.0044 (2)	−0.0042 (2)	0.0044 (3)
N1	0.0142 (3)	0.0112 (3)	0.0124 (3)	0.0017 (2)	0.0016 (2)	0.0031 (2)
C1	0.0126 (3)	0.0116 (3)	0.0126 (3)	0.0015 (2)	0.0012 (2)	0.0020 (2)
C2	0.0132 (3)	0.0103 (3)	0.0118 (3)	0.0020 (2)	−0.0002 (2)	0.0013 (2)
C3	0.0180 (3)	0.0117 (3)	0.0140 (3)	0.0042 (3)	−0.0018 (3)	0.0020 (2)
C4	0.0176 (4)	0.0132 (3)	0.0166 (3)	0.0058 (3)	−0.0025 (3)	0.0003 (3)
C5	0.0129 (3)	0.0133 (3)	0.0170 (3)	0.0039 (2)	−0.0002 (3)	−0.0016 (3)
C6	0.0128 (3)	0.0118 (3)	0.0146 (3)	0.0025 (2)	0.0011 (2)	0.0005 (2)
C7	0.0116 (3)	0.0100 (3)	0.0119 (3)	0.0021 (2)	0.0001 (2)	0.0011 (2)
C8	0.0113 (3)	0.0095 (3)	0.0116 (3)	0.0012 (2)	0.0002 (2)	0.0018 (2)
C9	0.0116 (3)	0.0108 (3)	0.0122 (3)	0.0020 (2)	0.0011 (2)	0.0024 (2)
C10	0.0122 (3)	0.0104 (3)	0.0116 (3)	0.0023 (2)	0.0016 (2)	0.0025 (2)
C11	0.0155 (3)	0.0150 (3)	0.0133 (3)	0.0014 (3)	−0.0009 (2)	0.0024 (3)
C12	0.0191 (4)	0.0187 (4)	0.0126 (3)	0.0050 (3)	0.0013 (3)	0.0038 (3)
C13	0.0195 (4)	0.0149 (3)	0.0159 (3)	0.0057 (3)	0.0063 (3)	0.0054 (3)
C14	0.0170 (4)	0.0140 (3)	0.0180 (3)	−0.0002 (3)	0.0045 (3)	0.0033 (3)
C15	0.0143 (3)	0.0141 (3)	0.0144 (3)	−0.0002 (3)	0.0010 (2)	0.0022 (2)
C16	0.0132 (3)	0.0136 (3)	0.0146 (3)	0.0042 (2)	0.0033 (2)	0.0042 (2)
C17	0.0308 (5)	0.0161 (4)	0.0229 (4)	0.0101 (3)	−0.0037 (4)	−0.0030 (3)
C18	0.0164 (4)	0.0185 (4)	0.0171 (3)	0.0039 (3)	0.0060 (3)	0.0045 (3)
Cl2	0.01584 (9)	0.01583 (9)	0.01397 (9)	0.00038 (6)	−0.00013 (6)	0.00351 (6)
O2	0.0187 (3)	0.0142 (3)	0.0297 (4)	0.0028 (2)	−0.0018 (3)	0.0094 (2)
O3	0.0148 (3)	0.0220 (3)	0.0209 (3)	−0.0037 (2)	−0.0018 (2)	0.0106 (2)
O4	0.0200 (3)	0.0196 (3)	0.0181 (3)	0.0001 (2)	−0.0077 (2)	0.0070 (2)
O5	0.0264 (4)	0.0343 (4)	0.0192 (3)	0.0083 (3)	0.0016 (3)	−0.0065 (3)

### Geometric parameters (Å, °)

**Table:**

Cl1—C5	1.7332 (9)	C10—C15	1.3976 (12)
O1—C16	1.2090 (11)	C11—C12	1.3946 (12)
N1—C1	1.3296 (11)	C11—H11A	1.025 (17)
N1—C2	1.3740 (11)	C12—C13	1.3903 (14)
N1—H1N1	0.829 (19)	C12—H12A	0.981 (17)
C1—C9	1.4123 (11)	C13—C14	1.3903 (13)
C1—C18	1.4919 (12)	C13—H13A	0.929 (16)
C2—C7	1.4097 (11)	C14—C15	1.3936 (12)
C2—C3	1.4115 (12)	C14—H14A	0.917 (17)
C3—C4	1.3754 (13)	C15—H15A	0.952 (16)
C3—H3A	0.954 (16)	C16—C17	1.4937 (14)
C4—C5	1.4117 (13)	C17—H17A	0.956 (19)
C4—H4A	0.926 (19)	C17—H17B	0.95 (2)
C5—C6	1.3738 (12)	C17—H17C	0.76 (2)
C6—C7	1.4159 (11)	C18—H18A	0.93 (2)
C6—H6A	0.942 (15)	C18—H18B	0.963 (19)
C7—C8	1.4295 (11)	C18—H18C	0.93 (2)
C8—C9	1.3788 (11)	Cl2—O5	1.4344 (8)
C8—C10	1.4864 (11)	Cl2—O3	1.4583 (7)
C9—C16	1.5200 (12)	Cl2—O2	1.4846 (7)
C10—C11	1.3965 (11)	Cl2—O4	1.5512 (7)
			
C1—N1—C2	123.82 (7)	C10—C11—H11A	121.2 (9)
C1—N1—H1N1	118.1 (13)	C13—C12—C11	120.12 (8)
C2—N1—H1N1	117.9 (13)	C13—C12—H12A	120.6 (10)
N1—C1—C9	118.77 (7)	C11—C12—H12A	119.3 (10)
N1—C1—C18	118.45 (7)	C12—C13—C14	119.96 (8)
C9—C1—C18	122.77 (8)	C12—C13—H13A	123.8 (10)
N1—C2—C7	118.94 (7)	C14—C13—H13A	116.1 (10)
N1—C2—C3	119.61 (7)	C13—C14—C15	120.52 (8)
C7—C2—C3	121.45 (8)	C13—C14—H14A	120.5 (10)
C4—C3—C2	118.80 (8)	C15—C14—H14A	119.0 (10)
C4—C3—H3A	120.4 (10)	C14—C15—C10	119.40 (8)
C2—C3—H3A	120.8 (10)	C14—C15—H15A	123.0 (10)
C3—C4—C5	119.88 (8)	C10—C15—H15A	117.5 (10)
C3—C4—H4A	121.9 (11)	O1—C16—C17	123.74 (8)
C5—C4—H4A	118.2 (11)	O1—C16—C9	119.76 (8)
C6—C5—C4	122.16 (8)	C17—C16—C9	116.48 (8)
C6—C5—Cl1	119.75 (7)	C16—C17—H17A	106.1 (11)
C4—C5—Cl1	118.09 (7)	C16—C17—H17B	105.6 (12)
C5—C6—C7	118.88 (8)	H17A—C17—H17B	114.6 (16)
C5—C6—H6A	119.5 (10)	C16—C17—H17C	112.6 (17)
C7—C6—H6A	121.6 (10)	H17A—C17—H17C	111 (2)
C2—C7—C6	118.79 (7)	H17B—C17—H17C	107 (2)
C2—C7—C8	118.43 (7)	C1—C18—H18A	110.3 (13)
C6—C7—C8	122.77 (7)	C1—C18—H18B	115.2 (11)
C9—C8—C7	119.31 (7)	H18A—C18—H18B	101.8 (17)
C9—C8—C10	121.17 (7)	C1—C18—H18C	106.8 (13)
C7—C8—C10	119.52 (7)	H18A—C18—H18C	115.6 (17)
C8—C9—C1	120.67 (7)	H18B—C18—H18C	107.4 (16)
C8—C9—C16	120.14 (7)	O5—Cl2—O3	114.23 (5)
C1—C9—C16	119.18 (7)	O5—Cl2—O2	111.92 (5)
C11—C10—C15	120.21 (7)	O3—Cl2—O2	110.06 (5)
C11—C10—C8	120.13 (7)	O5—Cl2—O4	109.31 (5)
C15—C10—C8	119.65 (7)	O3—Cl2—O4	104.17 (4)
C12—C11—C10	119.78 (8)	O2—Cl2—O4	106.60 (4)
C12—C11—H11A	119.0 (9)		
			
C2—N1—C1—C9	1.85 (12)	C7—C8—C9—C16	179.58 (7)
C2—N1—C1—C18	−177.33 (8)	C10—C8—C9—C16	−0.34 (12)
C1—N1—C2—C7	0.14 (12)	N1—C1—C9—C8	−2.26 (12)
C1—N1—C2—C3	−179.90 (8)	C18—C1—C9—C8	176.88 (8)
N1—C2—C3—C4	−178.83 (8)	N1—C1—C9—C16	178.84 (7)
C7—C2—C3—C4	1.12 (13)	C18—C1—C9—C16	−2.01 (12)
C2—C3—C4—C5	0.44 (13)	C9—C8—C10—C11	−78.54 (10)
C3—C4—C5—C6	−1.37 (13)	C7—C8—C10—C11	101.54 (10)
C3—C4—C5—Cl1	178.54 (7)	C9—C8—C10—C15	102.80 (10)
C4—C5—C6—C7	0.70 (13)	C7—C8—C10—C15	−77.12 (10)
Cl1—C5—C6—C7	−179.20 (6)	C15—C10—C11—C12	0.67 (13)
N1—C2—C7—C6	178.19 (7)	C8—C10—C11—C12	−177.97 (8)
C3—C2—C7—C6	−1.77 (12)	C10—C11—C12—C13	−1.27 (14)
N1—C2—C7—C8	−1.72 (11)	C11—C12—C13—C14	0.51 (14)
C3—C2—C7—C8	178.32 (7)	C12—C13—C14—C15	0.86 (14)
C5—C6—C7—C2	0.84 (12)	C13—C14—C15—C10	−1.44 (14)
C5—C6—C7—C8	−179.25 (8)	C11—C10—C15—C14	0.67 (13)
C2—C7—C8—C9	1.28 (11)	C8—C10—C15—C14	179.32 (8)
C6—C7—C8—C9	−178.63 (7)	C8—C9—C16—O1	89.51 (11)
C2—C7—C8—C10	−178.80 (7)	C1—C9—C16—O1	−91.59 (10)
C6—C7—C8—C10	1.30 (12)	C8—C9—C16—C17	−88.92 (10)
C7—C8—C9—C1	0.70 (12)	C1—C9—C16—C17	89.98 (10)
C10—C8—C9—C1	−179.22 (7)		

### Hydrogen-bond geometry (Å, °)

**Table:**

*D*—H···*A*	*D*—H	H···*A*	*D*···*A*	*D*—H···*A*
N1—H1N1···O3^i^	0.832 (18)	1.896 (18)	2.7177 (10)	169 (2)
C3—H3A···O2^ii^	0.955 (16)	2.583 (16)	3.3010 (11)	132.2 (12)
C15—H15A···O5	0.951 (16)	2.512 (16)	3.3716 (12)	150.4 (13)
C18—H18B···O5^iii^	0.97 (2)	2.53 (2)	3.3266 (13)	139.5 (14)
C12—H12A···Cg1^iv^	0.981 (17)	2.694 (17)	3.5810 (10)	150.6 (13)

Symmetry codes: (i) −*x*, −*y*, −*z*+1; (ii) *x*, *y*−1, *z*; (iii) *x*−1, *y*, *z*; (iv) −*x*, −*y*, −*z*.
